# Maternal Diets in India: Gaps, Barriers, and Opportunities

**DOI:** 10.3390/nu13103534

**Published:** 2021-10-09

**Authors:** Phuong Hong Nguyen, Shivani Kachwaha, Lan Mai Tran, Tina Sanghvi, Sebanti Ghosh, Bharati Kulkarni, Kalpana Beesabathuni, Purnima Menon, Vani Sethi

**Affiliations:** 1 Poverty, Health and Nutrition Division, International Food Policy Research Institute, Washington, DC 20005, USA; skachwa1@jhu.edu (S.K.); P.Menon@cgiar.org (P.M.); 2 Emory University, Atlanta, GA 30322, USA; Lantran2@emory.edu; 3 Alive & Thrive Initiative, FHI Solutions, Washington, DC 20009, USA; tsanghvi@fhi360.org (T.S.); sghosh@fhi360.org (S.G.); 4 ICMR-National Institute of Nutrition, Hyderabad 500007, India; dr.bharatikulkarni@gmail.com; 5 Sight and Life, New Delhi 500007, India; kalpana.beesabathuni@sightandlife.org; 6 UNICEF, New Delhi 110003, India; vsethi@unicef.org

**Keywords:** maternal nutrition, India, dietary intake, food, pregnant women

## Abstract

Suboptimal dietary intake is a critical cause of poor maternal nutrition, with several adverse consequences both for mothers and for their children. This study aimed to (1) assess maternal dietary patterns in India; (2) examine enablers and barriers in adopting recommended diets; (3) review current policy and program strategies to improve dietary intakes. We used mixed methods, including empirical analysis, compiling data from available national and subnational surveys, and reviewing literature, policy, and program strategies. Diets among pregnant women are characterized by low energy, macronutrient imbalance, and inadequate micronutrient intake. Supply- and demand-side constraints to healthy diets include food unavailability, poor economic situation, low exposure to nutrition counselling, food restrictions and taboos, adverse family influence and gender norms, and gaps in knowledge. Intervention strategies with potential to improve maternal diets include food-based programs, behavior change communication, and nutrition-sensitive agriculture interventions. However, strategies face implementation bottlenecks and limited effectiveness in real-world at-scale impact evaluations. In conclusion, investments in systems approaches spanning health, nutrition, and agriculture sectors, with evaluation frameworks at subnational levels, are needed to promote healthy diets for women.

## 1. Introduction

Poor maternal nutrition before and during pregnancy is a significant public health concern due to adverse consequences both for mothers and for their children. Maternal malnutrition is associated with increased risk of maternal morbidity, preterm deliveries, and small-for-gestational-age babies [1,2]. Maternal undernutrition remains a global concern, with 24% of women in South Asia having low body mass index (BMI) [1]. Further, 30% of women of reproductive age and 37% of pregnant women are anemic [3]. Overweight/obesity among women is an emerging issue affecting most low–middle-income countries [4]. 

Adequate dietary quantity and quality is an established determinant of poor maternal nutrition. In 2017, the Global Burden of Disease Study found that poor diets caused more deaths than did all other risk factors, attributable to a total of 11 million deaths, with 3 million deaths each due to high sodium intake and low intake of whole grains, and 2 million deaths due to low intake of fruits [5]. Poor diet during pregnancy has been associated with adverse pregnancy outcomes [2], which, in turn, increase child susceptibility to cardiometabolic diseases in later life [6]. Despite this evidence, maternal diets in low- and middle-income countries (LMICs) suffer from macronutrient and micronutrient imbalance, and are predominantly plant-based [7]. Many women have concurrent deficiencies of essential micronutrients such as iron, zinc, vitamins B12 and D, and iodine through their reproductive years [1]. A study from Guatemala, India, Pakistan, and Democratic Republic of the Congo reported that more than 80% of women had inadequate intakes of essential micronutrients [8].

In India, more than half of women are anemic [9], nearly 40% of women 15–49 years are underweight [10], and nearly 25% of women are overweight/obese [10]. Cereals and millets form the bulk of rural diets, as indicated by the National Nutrition Monitoring Bureau (NNMB) surveys in 10 Indian states, with only about half of pregnant women consuming adequate quantities of protein and energy [11]. NNMB also showed that intakes of iron, vitamins A and C, and folic acid were less than 50% of recommended levels for most pregnant women. Suboptimal diets, together with other factors, including early and multiple pregnancies, poverty, caste discrimination, and gender inequality, contribute to poor maternal nutrition in India [12]. Increased marketing of unhealthy and cheap foods along with high carbohydrate and sugar in public procurement systems have contributed to rising overweight/obesity and other noncommunicable diseases. High rates of death and years of life lost due to ill health, disability, or early death related to diet were also reported in the 2017 Global Burden of Disease Study [5].

The World Health Organization (WHO) guidelines on antenatal care (ANC) recommend 49 interventions, of which 14 are nutrition interventions [13]. The national guidelines in India follow global recommendations on a package of essential nutrition interventions for pregnant women, among them, improving diets. India’s flagship National Nutrition Mission, POSHAN Abhiyaan, aims to improve maternal nutrition through capacity building, leveraging technology, behavior change communication, community mobilization, and cross-sectoral convergence [14]. Current intervention strategies in India to improve maternal diets include a rich portfolio of programs and policies that address dietary intakes through take-home rations and hot cooked meals for pregnant and lactating women, micronutrient supplements, food fortification, delivery of subsidized staples through the public distribution system, cash transfers, nutrition-sensitive agriculture, and diet education and counseling. However, weak delivery systems, logistical gaps, resource scarcity, poor utilization, and wide disparities in access to food and health resources continue to hamper progress in improving diets among pregnant women [12]. 

This paper aims to (1) assess maternal dietary patterns in India; (2) examine enablers and barriers in adopting recommended diets; (3) review current policy and program strategies in India to improve dietary intakes and assess their effectiveness. Findings from this study will highlight policy, program, and research gaps, and will be used to offer recommendations for future action.

## 2. Materials and Methods

We used mixed methods for the study, including (1) reviewing data from available national surveys on food and nutrient intakes among pregnant women; (2) examining data on dietary intakes from the latest available national representative surveys to document dietary patterns and identify determinants of dietary patterns; (3) conducting a literature review to identify enablers and barriers to adopting recommended diets; (4) reviewing policy and program strategies to improve dietary intakes and their effectiveness; (5) integrating insights to offer recommendations for future action.

### 2.1. Review of Dietary Data from National Surveys

At the national level, the NNMB was established in 1972 by the National Institute of Nutrition and Indian Council of Medical Research to collect data on food and nutrient intakes in rural and tribal populations across 10 states (Kerala, Tamil Nadu, Karnataka, Andhra Pradesh, Maharashtra, Gujarat, Madhya Pradesh, Orissa, West Bengal, and Uttar Pradesh). Data were collected approximately every 10 years, with the latest round available in 2012 [15]. A special survey was also conducted in 2015, but only for the urban population and among nonpregnant and nonlactating women. The 2012 survey included a 24-h dietary recall with a small sample of pregnant women (*n* = 322) to collect information on average food and nutrient intakes. We extracted relevant data from the report to summarize dietary intakes among pregnant women.

### 2.2. Empirical Analysis of Publicly Available Data

We used data from National Family Health Survey 2005–06 (NFHS-3) [16] and 2015–16 (NFHS-4) [17]. NFHS-3 included data from 109,041 households and was representative at the state level. NHFS-4 was representative at both state and district levels, with data from 601,509 households. This analysis used data from pregnant women (*n* = 38,339). Dietary intake was collected using self-reported information on the frequency (daily, weekly, occasionally, and never) of 9 food groups (milk or curd, pulses or beans, dark green leafy vegetables, fruits, eggs, fish, chicken or meat, fried foods, and aerated drinks). We first compared changes in daily food group consumption from 2006 to 2016. We then used maps to visualize state-level variation in food intake in 2016. To examine inequalities in dietary intake, we used equity plots disaggregated by residence, wealth quintile, and caste group. Finally, we performed multivariable regression analyses to assess the association between a select set of determinants and food group consumption. Because the proportion of women who consumed fish, meat, and eggs daily was very low, we used an indicator of daily consumption of any animal source foods. We were not be able to compute minimum dietary diversity for women due to limited available data. The variables selected for regression models include maternal age, early marriage (defined as age at first marriage <18 years), early birth (defined as age at first birth <19 years), education, caste status, occupation, decision-making power, and household socioeconomic status (SES). Household SES was constructed using a principal component extracted from multiple variables, including household ownership of fifteen assets. All analyses were performed using Stata version 16.1. All regression models were adjusted for the cluster sampling design and sampling weights used in the survey. 

### 2.3. Literature Review

The literature review aimed to (1) identify enablers and barriers in adopting recommended diets and (2) review policies/programs and assess the effectiveness of current intervention strategies to improve dietary intakes. Between 7 June and 17 July2021, we searched PubMed, MEDLINE, and Google Scholar with the following search terms ((Dietary intake OR diet OR Food consumption) AND (maternal OR pregnancy) AND (India)) with no language restriction but limited to “humans”, “Adolescent: 13–18 years”, “Adult: 19+ years”, and time between January 2000 and June 2021. In addition, we sourced grey literature from the last 10 years, including interventions, program evaluations, and unpublished literature from the government or organizational websites. The abstracts of all potential publications were reviewed independently by the first and second authors, then the full text of potentially eligible studies was obtained for in-depth review. We extracted data using a standardized template including authors, publication year, state, study objective, study design and sample size, enablers and/or barriers, and key findings of interventions. 

The literature review of key enablers and barriers influencing maternal dietary intakes identified 20 studies across 13 states, including from Tamil Nadu, Karnataka, West Bengal, Maharashtra, Andhra Pradesh, Sikkim, Uttar Pradesh, Delhi, Bihar, Rajasthan, Chhattisgarh, Odisha, and Madhya Pradesh. Most studies (*n* = 18) aimed to understand maternal dietary beliefs and practices, and two studies aimed to assess affordability and access to nutritious diets. Studies varied in design and methods and included qualitative ethnographic studies (in-depth interview and focus group discussion), quantitative cross-sectional surveys, and market surveys.

The literature review of interventions identified a total of 14 studies from 10 states, including Andhra Pradesh, Telangana, Karnataka, Odisha, Rajasthan, Punjab, Tamil Nadu, Haryana, Uttar Pradesh, and Kerala. Most studies aimed to assess the effects of different strategies to improve maternal nutrition and used various methods and study designs: cluster-randomized control trials, quasi-experimental studies, cross-sectional surveys, and qualitative methods (focused group discussions and in-depth interviews). 

## 3. Results

### 3.1. Current Situation of Maternal Diets in India

#### 3.1.1. Maternal Diets at the National Level

The review of NNMB data showed that energy and macronutrient intakes were lower than recommended (Table 1). The median intake of energy was 1736 Kcal [15], compared to the estimated average requirement (EAR) of 2010 Kcal, and the median intake of protein was 45 g, compared to the EAR of 54 g for sedentary pregnant women [18]; about half of pregnant women had inadequate intakes of energy, 35% had inadequate protein intake, and 20% had inadequate fat intake. Intakes of essential micronutrients such as iron, vitamin A, riboflavin, vitamin C, and folic acid were less than 50% of RDA among most pregnant women (~50–80%) [15]. The average intake of several food groups (cereals and millets, nuts and seeds, milk and milk products, and sugar) decreased over time between 1997 and 2012 among pregnant women. Daily intakes of all nutrients except vitamin A, thiamine, and niacin also declined from 1997 to 2012 (data not shown).

**Figure 1:**
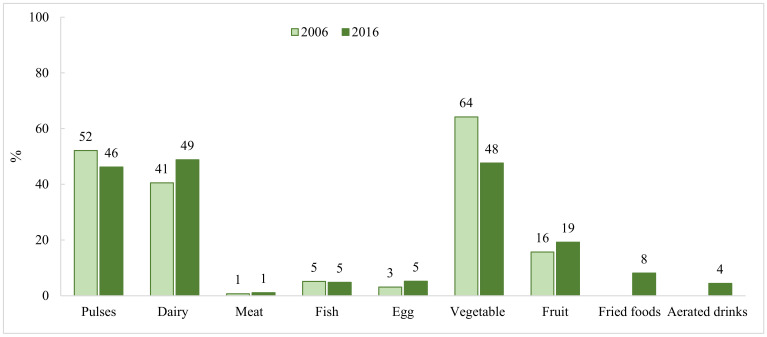
Dietary intake among pregnant women in India between 2006 and 2016 ^1^. ^1^ National data comes from National Family Health Surveys in 2005–2006 and 2015–2016.

Analysis of NFHS data revealed no improvement in dietary intakes among PW between 2006 and 2016, except for dairy, which increased marginally over time (8 percentage points, pp) (Figure 1). Consumption of pulses and vegetables reduced over time (6 and 16 pp, respectively). In 2016, diets among PW remained suboptimal for most food groups: less than 10% of PW consumed meat, fish, and/or eggs, a fifth consumed fruits, and about half consumed pulses, dairy, and vegetables daily. Unhealthy food consumption including aerated beverages and fried foods was 4–8% among PW in 2016.

**Figure 2:**
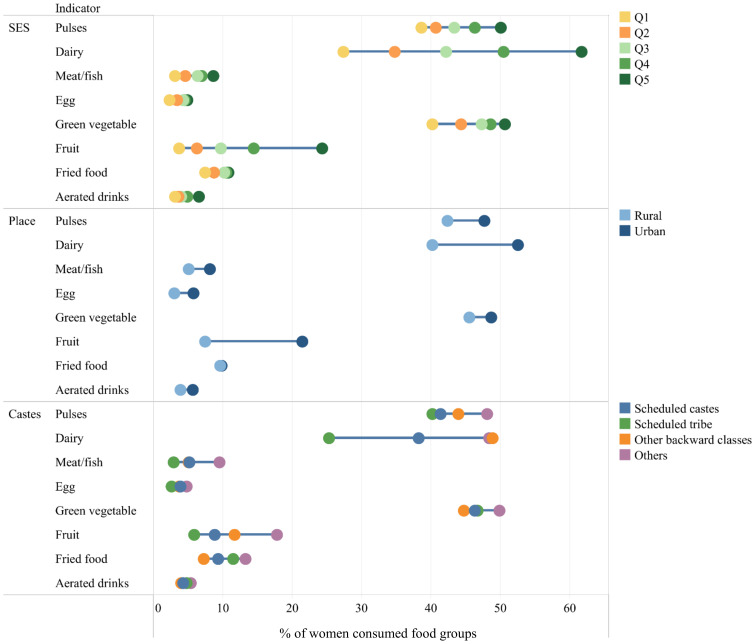
Socioeconomic ^1^, residential, and caste inequality in dietary intake among Indian pregnant women. SES: socioeconomic status; Q: quintile. Data are from National Family Health Survey 2015–2016. ^1^ Household SES was constructed using the principal component analysis method. The first principal component was used to divide household SES into quintiles; the lowest quintile (Q1) represented the poorest 20% of the population and the highest quintile (Q5) represented the richest 20%.

Food group consumption was higher among wealthier compared with poorer households, with the highest gap being in dairy consumption (35 percentage points, pp), followed by fruits (21 pp), pulses (12 pp), and green vegetables (11 pp) (Figure 2). Similar inequity in dietary intakes was observed between residential areas and among caste groups; women living in rural areas or belonging to backward castes had lower intakes compared with their counterparts.

#### 3.1.2. Maternal Diets at the Subnational Level

**Figure 3:**
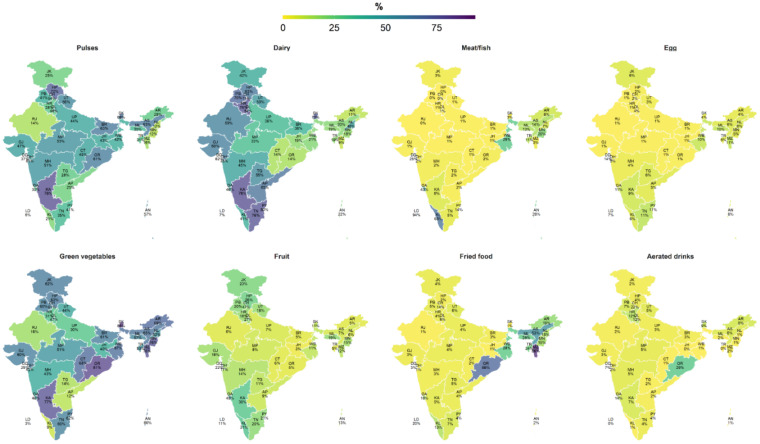
Subnational variation in dietary intake among pregnant women in India ^1^. ^1^ Data comes from the National Family Health Survey in 2015–2016. Values are percentages of women consuming these food groups. AN: Andaman and Nicobar Island; AP: Andhra Pradesh; AR: Arunachal Pradesh; AS: Assam; BR: Bihar; CH: Chandigarh; CT: Chhattisgarh; DD: Daman and Diu; DH: Dadara and Nagar Havelli; DL: NCT of Delhi; GA: Goa; GJ: Gujarat; HP: Himachal Pradesh; HR: Haryana; JH: Jharkhand; JK: Jammu and Kashmir; KA: Karnataka; KL: Kerala; LD: Lakshadweep; MH: Maharashtra; ML: Meghalaya; MN: Manipur; MP: Madhya Pradesh; MZ: Mizoram; NL: Nagaland; OR: Odisha; PB: Punjab; PY: Puducherry; RJ: Rajasthan; SK: Sikkim; TG: Telangana; TN: Tamil Nadu; TR: Tripura; UP: Uttar Pradesh; UT: Uttarakhand; WB: West Bengal.

Subnational data on dietary intake showed a low percent of PW consuming animal-source foods in most states in 2015–2016 (<30%), with the exception of Kerala where 65% of women consumed meat or fish (Figure 3). Similarly, low consumption of fruits was observed for most states. There were substantial subnational variations across states in consumption of pulses, dairy, and green vegetables (ranging from 3% to 80%). Consumption of unhealthy foods was higher in some Eastern states, particularly Odisha (69%) and Mizoram (86%). 

Subnational data on dietary intake among PW based on 24-h recall of 10 food groups were available for some states (Uttar Pradesh [21], Bihar, Odisha, and Chhattisgarh [22] (Figure S1). In Uttar Pradesh, only about a fifth of PW achieved dietary diversity (consumed ≥5 food groups in a day), while about third of PW in Bihar and more than half of PW in Odisha and Chhattisgarh consumed diverse diets. 

Data on macro- and micronutrient intakes at the subnational level were available in Uttar Pradesh only [21] (Table 1). Energy intakes were lower than recommended, and macronutrient intakes were imbalanced. Per capita mean energy intake was 1759 kcal in Uttar Pradesh, far below the individual requirement of 2322 kcal/day. Over 75% of energy came from carbohydrates (recommendation: 45–65%), 12% from protein (recommendation: 10–35%), and 11% from fat (recommendation: 20–35%). In addition, >80% of PW had inadequate intakes for 9 of 11 essential micronutrients. 

### 3.2. Enablers of and Barriers to Adopting Recommended Diets in India

Key enablers of and barriers to maternal diets identified through empirical analysis (Table 2) and the literature review (Table 3) are presented by theme below.

#### 3.2.1. Food Availability and Accessibility

The review found mixed results on the role of food availability and accessibility in influencing maternal diets. Findings from an ethnographic study in Tamil Nadu found that women reduced consumption of millets and switched to rice due to lack of availability [28]. Another study in Maharashtra similarly found that with easier food access, consumption of staple foods reduced and of other foods increased [29]. In addition, qualitative in-depth interviews in Uttar Pradesh showed that pregnant women were constrained by limited food items in their markets and travel limitations for women [30]. In contrast, a cost-of-diet survey found that food availability in markets was not a key barrier to nutritious diets [31], but other factors that may affect availability, such as seasonality and market access, were not reported.

#### 3.2.2. Economic Constraint and Affordability

Both the empirical analysis and the literature review highlighted the important role of higher economic status as an enabler and financial constraints as a barrier in adopting recommended diets. Regression analysis showed that, compared to the poorest households, the richest households were 3.7 times more likely to consume dairy, nearly twice as likely to consume animal-source foods, and 5 times more likely to consume dark green leafy vegetables. Larger households (≥7 people) were less likely to consume animal-source foods and dark green leafy vegetables, but more likely to consume pulses.

Findings from studies identified during the literature review corroborated findings from the empirical analysis [21,22,24,30,31,32,35,36]. Mothers belonging to richer households were about two times more likely to consume a diverse diet (≥5 food groups) [21,36]. Nutritious diets were unaffordable for ~65–75% of households [31,35]. Women did not consume recommended foods due to lack of money [38]. Higher parity was associated with less consumption of special foods during pregnancy, such as milk, animal protein, pulses, and fruits [34], and was associated with lower diet diversity [21,34,36].

#### 3.2.3. Exposure to Health and Nutrition Services

Receipt of health and nutrition services and counseling on diet was associated with a 1.5–3 times greater likelihood of higher dietary diversity and consuming more food groups [21,39,40]. Dietary diversity was lower among women who had no prior contact with a health professional (OR = 0.6) [36]. In addition, interventions of home production, subsidized grains, and food supplementation improved the affordability of nutritious diets by 10–35% [31]. Although overall use of food supplementation increased between 2006 and 2016 from 19% to 53%, marginalized groups such as lower castes and tribes, the poorest quintiles, or those with low schooling levels were still left behind [27]. 

#### 3.2.4. Maternal Education

In our empirical analysis, compared to illiterate mothers, those with high school or higher education were nearly twice as likely to consume pulses, 3 times more likely to consume dairy and animal source foods, and 5.6 times more likely to consume dark green leafy vegetables.

Studies from the literature review showed that mothers with higher education or at least 6 years of education were more likely to have higher dietary diversity compared with illiterate mothers [21,36]. Higher literacy was associated with consuming special foods such as milk and animal source foods during pregnancy [34]. 

#### 3.2.5. Maternal Knowledge

The review found that higher maternal knowledge was associated with higher diet diversity (OR = 2.2) and greater number of food groups consumed [21]. Qualitative interviews also confirmed that high awareness and high knowledge of diet diversity recommendations were the key enablers affecting behavioral adoption for diet diversity [30]. Additional enablers included knowing that nutritious foods included fruits, vegetables, egg, and animal-source foods; 60% of women believed that these foods were beneficial for health and should be consumed during pregnancy [28,32,33].

#### 3.2.6. Food Taboos and Restrictions during Pregnancy

Food taboos and restrictions during pregnancy were mentioned in several qualitative studies across different states [24,25,26,28,33,34,38]. Most mothers (65% to 82%) believed that some food items should be restricted during pregnancy [33,34]. Various fruits (banana, papaya, jackfruit, and coconut), vegetables (brinjal and leafy vegetables), meat, fish, and eggs were believed to be heat-producing or sour, thus should be restricted during pregnancy to prevent miscarriage and fetal malformations, and promote easy delivery [25,26,28,38]. Other beliefs associated with the type of food given to pregnant women included “casting an evil eye” or “color of the baby” [25]. Most food avoidance was based on advice from elders and family members [28]. Mothers who had strong likes and dislikes during pregnancy and experienced nausea were less likely to follow diet recommendations [30].

#### 3.2.7. Family Influence

The review yielded mixed findings on the role of family members in influencing maternal diets. Qualitative in-depth interviews in one study showed that family members had high levels of awareness and motivation to ensure nutritious diets for pregnant women [30]. Specifically, husbands supported pregnant women by procuring nutritious foods from the market and reminding them to eat recommended foods. In contrast, another study reported that family elders restricted certain foods during pregnancy [28]. 

#### 3.2.8. Gender Norms

Strong gendered social norms affected many aspects of women’s health and well-being, including nutrition behavior and practices [37]. From childhood to adulthood, women’s health and nutrition is given lower priority than is men’s health and nutrition. Women are usually the last to eat in the household and eat the least amount of food [37]. In many households, men were responsible for procurement of food from the market, often themselves deciding what food items to buy or buying food items as decided by the mother-in-law while women had limited access to markets and minimal ability in exercising their choices for food items [23].

#### 3.2.9. Other Demographic Factors

Our regression analysis showed that households in urban areas were 1.7 times more likely to consume dairy and 2.7 times more likely to consume dark green leafy vegetables than were those in rural areas. Belonging to scheduled caste/tribe or other backward classes was associated with lower consumption of pulses (OR = 0.85) and dairy (OR = 0.79). Early marriage was associated with 17% and 32% lower consumption of dairy and dark green leafy vegetables, respectively. The literature review confirmed that women belonging to lower caste (OR = 0.9) and rural households (OR = 6.9) were more likely to have lower diversity [21,39,40].

#### 3.2.10. Indigenous Foods

Trends in the use of indigenous foods also affect PW’s diets. Being a country with the largest tribal population in the world, India is composed of 705 distinct tribal groups who make up nearly one-tenth of the country’s population (104 million). Tribal people cultivate local foods and collect uncultivated foods from forests; thus, they are able to eat nutritious food [41]. However, several barriers affecting this population included experiencing discrimination in access to agricultural inputs or economic opportunities, expulsion from their lands, and displacement to urban settlements where they lose access to their cultural heritage and their traditional foods, practices, and languages [42]. The introduction of modern technologies has improved their lives but has also brought other challenges, including altering the nutritive value of indigenous foods due to chemical use, or reducing diversity of traditional foods through degradation of forest and agro-biodiversity [43].

### 3.3. Policies to Improve Maternal Diet

**Figure 4:**
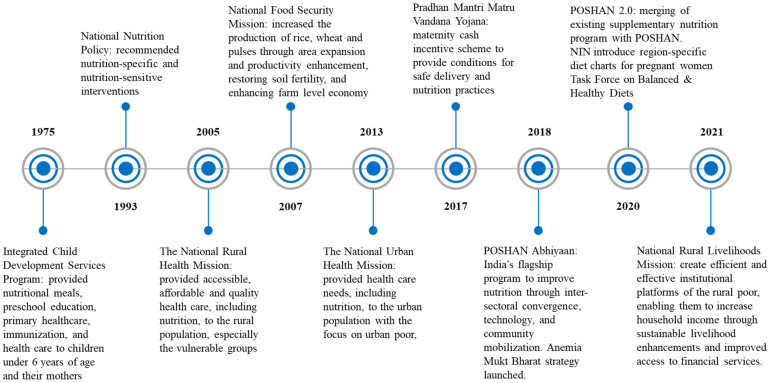
Summary of key policies to improve maternal nutrition and maternal diets.

Selected policies to improve maternal diet are summarized in Figure 4. The National Nutrition Policy (NNP) 1993, formulated in the national development context, positioned nutrition as a complex development problem linked to agriculture, food production, and poverty. The NNP recommended both direct and indirect nutrition interventions that include nutrition-specific and nutrition-sensitive interventions. India’s nutrition policies recognize the multifaceted nature of interventions necessary to accelerate progress in nutrition. Various programs and schemes have been launched and expanded over the years to improve maternal diets and nutritional status. Schemes in India are largely delivered through flagship programs of two ministries: (1) the Integrated Child Development Services (ICDS) scheme under the Ministry of Women and Child Development provides micronutrient-fortified supplementary food and/or energy-dense take-home rations for pregnant women and breast-feeding mothers; (2) the Ministry of Health and Family Welfare delivers micronutrient supplements (IFA and calcium), deworming, weight gain monitoring, and dietary counseling to pregnant women as part of antenatal care services.

One of the notable current programs is POSHAN Abhiyaan, (Prime Minister’s overarching scheme for holistic nutrition), India’s flagship program that was launched in 2018, with an aim to improve nutritional outcomes for children, pregnant women, and lactating mothers. The core components of the POSHAN Abhiyan include a focus on inter-sectoral convergence, use of technology, and *Jan Andolan* (people’s movement). The current program is being strengthened in the form of POSHAN 2.0, where an existing Supplementary Nutrition Program and the POSHAN Abhiyan will be merged to increase nutritional content, delivery, outreach, and outcomes. Another important program is a maternity benefit program titled “Pradhan Mantri Matru Vandana Yojana (PMMVY)”. The cash incentive helps to improve safe delivery and good nutrition and feeding practices. Other programs, including the National Health Mission, Anemia Mukt Bharat program, National Rural Livelihoods Mission, and Task Force on Healthy and Balanced Diets, also indirectly or directly aim to improve maternal nutrition. However, despite evidence of rising overweight and obesity among women, the current policy guidelines lack specific recommendations on improving diets for pregnant women in different body mass index (BMI) categories ranging from thin to obese. In 2020, the Ministry of Women and Child Development started sharing region-specific diet charts, which were prepared by Indian Council for Medical Research—National Institute of Nutrition [44]. These charts have been created for six regions—North, South Central, East, West, and North East—to guide pregnant women on their nutritional requirements, keeping different dietary habits and cultures in mind. The current implementation status of the region-specific diet chart is unknown. 

### 3.4. Current Intervention Strategies in India to Improve Dietary Intakes and Their Effectiveness

Several intervention strategies have been implemented to improve maternal diets in India, including hot cooked meals, food innovations in take-home rations (THR), maternal cash transfer programs, food fortification of staples, behavior change communication (BCC) strategies, and nutrition-sensitive agriculture interventions (Table 4). 

#### 3.4.1. Hot Cooked Meals

The review identified three studies on hot cooked meals, which found some improvements in the proportion of women consuming diverse diets post-intervention (~60%), consumption of specific food groups (eggs and milk—74% to 96%), and energy and protein intake among pregnant women [45,46,47]. However, several implementation issues were noted, including lower distribution of foods and fewer feeding days compared with the program protocol. 

#### 3.4.2. Food Innovations in Take-Home Rations

Three studies were found on food innovations that are applicable for THR through centralized models of production. An innovative enzyme called phytase was found to significantly improve mineral bioavailability from plant-based diets and cereal–legume foods [60,61]. Another area for innovation in THR is the role of fermented foods on gut health and nutrition status. A *Lactobacillus plantarum* strain isolated from the *dosa* has been found to inhibit the growth of a range of food-borne pathogens [48]. 

#### 3.4.3. Maternal Cash Transfers

Only one study was found on maternal cash transfers, which reported a decline of 0.84 on the household food insecurity access scale following receipt of cash transfer [49]. The key bottlenecks were smaller transfers than expected and delays in payment to beneficiaries. 

#### 3.4.4. Food Fortification of Staples

Two studies were identified on fortification of staples, which reported low levels of fortification of wheat flour (6%), edible oil (24%), and salt (66%) [50,51]. Key challenges related to food fortification were low demand given low consumption of wheat in many predominantly rice-eating regions in the South and most households purchasing whole grain and milling produce locally (82%) in the North. The other barrier was lack of monitoring of compliance to fortification protocols.

#### 3.4.5. Behavior Change Communication

Strategies of BCC were reported in six studies, which included nutrition counseling and visual tools to improve maternal diets. Most studies reported positive impacts on dietary intake, including increased quantity, frequency (from 2–3 to 3–4 meals), and quality of diets (increased intake of vitamin A-rich fruits, other vegetables, and higher protein intake), however few studies reported impacts on overall dietary diversity [52,53,54,55,56]. Despite improvements, dietary intakes did not meet recommended levels for most micronutrients. The key factors associated with adoption of recommended behaviors were maternal perceptions, role of family members, food preferences, and limited resources. Many research gaps remain, however, on evidence of BCC complemented by systems strengthening efforts to improve quality and sustainability. The Alive & Thrive maternal nutrition study is the only study that included evidence on integrating multiple strategies as part of BCC interventions in existing antenatal care [54]. Other studies documented the development and feasibility of integrating a maternal service package for mothers of children with severe acute malnutrition admitted to nutrition rehabilitation centers in Delhi and Uttar Pradesh [57,62]. 

#### 3.4.6. Nutrition-Sensitive Agriculture Interventions: Empowerment through Income and Access to Nutritious Foods

A model of improving socioeconomic and nutrition conditions through women’s empowerment is a poultry cooperative in central India—Madhya Pradesh Women Poultry Producers’ Company Limited (MPWPCL) [58]. MPWPCL is an all-women-owned and -managed farmer co-operative that promotes smallholder poultry women farmers in difficult-to-reach areas, by aggregating them in clusters. Based on market principles, MPWCL generates wealth leading to their socioeconomic upliftment. Women are trained in poultry farming and provided capital to set up small farms. All farms are supported with high-quality and a steady supply of affordable inputs, with regular supervision. By doing so, good poultry management practices are reinforced, resulting in improved farm operational efficiency and safety from disease outbreaks. As a result, income increased by eight times per household and more eggs became available in the community. Another study reported on nutrition-sensitive agriculture interventions and found positive impacts on maternal dietary diversity through exposure to videos and participatory learning activities [59].

## 4. Discussion

Maternal diets affect women’s immediate well-being and livelihoods as well as the health, cognition, and productivity of the next generation [1]. Through literature review and empirical analysis with multiple data sources, this mixed methods study assessed trends in maternal diets in India, identified enablers of and barriers to adopting diets, and documented program interventions and policies in India. Dietary intakes among pregnant women were suboptimal with low energy, imbalanced macronutrients, and inadequate micronutrient intakes, and varied substantially across states. Economic constraints, food taboos, and social norms play an important role, with family members emerging as key influential persons. Interventions that can improve maternal diets in India are not well evaluated but highlight the importance of ICDS food-based programs, BCC strategies, and nutrition-sensitive agriculture interventions. 

### 4.1. Data Gaps on Maternal Dietary Practices and Evaluation Impacts of Programs and Interventions

This study highlighted critical data gaps regarding maternal dietary practices and evaluation impacts of programs and interventions. The Global Nutrition Report in 2017 recognized the importance of high-quality, regular, disaggregated data to ensure accountability and progress towards improving nutrition universally [63]. However, nationally representative data on maternal diets in India are very limited. The most recent NFHS data collected dietary information using food frequency questions that did not include sufficient food groups to calculate maternal dietary diversity, macro- or micronutrient intakes, or food security status to help understand consumption patterns of families affected by food insecurity. The NNMB data used a 24-h dietary recall of food and nutrient intakes but was limited by a small sample of pregnant women in rural areas (*n* = 322 for all 10 states), and the most recent round available is from 2012. The NNMB data collected in urban areas in 2015–2016 did not include data for pregnant women [64]. Although fish is recommended as a source of essential nutrients during pregnancy in the national guideline [65], we did not identify any studies that specifically evaluated meat or fish separately, nor any that reported on omega-3 or omega-6 fatty acids. The evidence review also found limited available research on impacts of programs and interventions. For example, although food supplementation under ICDS scheme has been widely implemented, only a few studies exist on the hot cooked meal and THR program. These small-scale studies mainly focus on implementation issues and lack impact evaluation to assess the extent of the program’s impact on maternal nutrition outcomes [66]. Rigorous evaluations are needed to test successful innovations and models to harmonize THR recipes and standards across all states. 

### 4.2. Factors Influencing Maternal Diets in India

Multiple supply- and demand-side factors influence maternal diets in India. The predominant factors include food unavailability, economic constraints, low exposure to nutrition services and counselling, food restrictions and taboos during pregnancy, family influence, gender norms, and gaps in maternal knowledge. There is, however, limited information on how household budgets can be adjusted to procure specific nutrient-rich foods for meeting the needs of pregnant women. The evaluations in India identified intervention-specific limitations in implementation, such as inadequate food distributed and fewer days of distribution in hot meals programs, small amounts and delayed payments in cash transfer programs, and low coverage of foods selected for fortification and inadequate fortification levels. Thus, investments to improve nutrition, including maternal diet, must be fundamentally multisectoral in nature and focus on strengthening the delivery, reach, and utilization of nutrition-specific interventions. 

### 4.3. Intervention Strategies with Potential to Improve Maternal Diets in India

The literature review also found few intervention strategies with potential to improve maternal diets in India. Specifically, food supplementation and cash transfer program evaluations suggest improvements in affordability of diets by reducing economic constraints in obtaining recommended foods [67]. Lack of food availability is a complex problem with many potential factors, including access to markets, diversity and types of food available in markets, low agriculture production, postharvest losses, and seasonality. Strategies to improve food availability include kitchen gardens, homestead production, women cooperatives, and nutrition-sensitive agriculture interventions [68]. Food systems approach (from Farm to Fork) needs to be strengthened to ensure affordability, availability, accessibility, food security, sustainability, and resilience in production and promotion of diverse nutrient-rich foods and safe diets for pregnant women. 

BCC, along with community sensitization and engagement, can be an effective strategy to improve maternal knowledge, motivate family members (especially husbands and mothers-in-law) to support maternal diets, and clarify misconceptions about food taboos during pregnancy where foods are available but not utilized optimally [69]. BCC needs to be complemented by efforts to address the gender-based power differentials within the family related to decision making on choice of food items, procurement, preparation, and food distribution, and efforts to improve women’s informed decision-making power, agency, and access to resources. Multiple platforms (at home, community, and facility levels) across multiple sectors (health, nutrition, rural development/self-help groups) need to be engaged to ensure coverage, intensity, continuity, and quality. Experience from the maternal nutrition study in Uttar Pradesh, India [54] and Bangladesh [69] highlighted the crucial roles of investing in systems strengthening efforts of capacity building (knowledge and skills for counselling, and supportive supervision of frontline workers and providers across multiple sectors), use of data to review and track progress, setting up of convergent action planning and review mechanisms, and ensuring accountability of program managers. Regular mass media campaigns using existing social media platforms of all stakeholders are needed to disseminate accurate and uniform information about the importance of healthy and balanced diets for pregnant women. 

Over the past two decades, there have been several experiments in the THR program. Dedicated government bodies, frontline workers, and supporting organizations have led to better recipes, viable production models, and mechanisms for women’s participation, as well as better governance and accountability. The initiatives have been further strengthened by the Supreme Court’s ruling emphasizing quality and safety standards during THR production and the role of automated machines for assuring food safety and quality. Fermented food has emerged as an innovation in THR. While there is limited evidence on the health benefits of fermented foods during pregnancy, India is among very few countries that encourage the consumption of fermented foods during pregnancy in its national food guide [70]. There are more than 350 types of fermented foods in India [71], and these foods are common in every food culture—modern or traditional; rural or urban or tribal. They can be prepared at home or through self-help groups and are convenient to produce through the decentralized THR production models. It would be worth exploring the effect of fermented foods on maternal health.

### 4.4. Policy Implication

Our findings highlight policy, program, and research gaps, and offer recommendations for future action. Policy and program guidelines across multiple health, nutrition, and agriculture sectors need to prioritize and invest sufficiently in resource allocation towards accelerating efforts to improve maternal diets. Operational guidelines need to be strengthened and updated. Guidelines should include micronutrient supplement formulations, locally adapted food supplements, the required number of counseling sessions and counseling guidelines on diet, and management of special dietary requirements for at-risk groups of pregnant women, including overweight and obese pregnant women, and should clarify roles among functionaries across different sectors. Strategies need to be both universal and targeted to address special requirements and must be contextualized to local environments to integrate local food and diet patterns and sociocultural norms around dietary practices of pregnant women. Emphasis should be on combined packages across health and food systems, social safety nets, and women’s empowerment with social and behavior change integral to all interventions.

## 5. Conclusions

Poor diets in pregnancy have wide-ranging and long-lasting detrimental impacts [1,2]. Investments are urgently needed in ongoing tracking of dietary patterns of PW, investigating factors influencing diets, addressing barriers through interventions, and development of quality standards for implementing programs. Priority should be placed on commitment to research for closing data gaps and strengthening existing platforms reaching pregnant women in all socioeconomic classes. Social behavior change interventions should be developed and adapted for specific locations as a part of existing programs. Other broader programs should be considered, including strengthening an ANC package, community mobilization for empowering women through self-help groups, and ICDS food-based programs combined with community awareness.

## Figures and Tables

**Table 1:** Macro- and micronutrient intakes.

	NNMB Survey ^1^ (*n* = 322)			Uttar Pradesh ^2^ (*n* = 667)		
	Average Intake per Day		Inadequate Intake ^3^	Average Intake per Day		Inadequate Intake ^4^
	Median	Mean (SD)	%	Median	Mean (SD)	%
**Macronutrients**						
Energy intake (kcal)	1736	1773 (604)	13.7	1647	1759 (743)	NA
Protein intake (g)	44.5	48.6 (21.5)	35.7	55.8	59.4 (28.3)	10.9
Fat (g)	23.5	28.1 (17.5)	19.6	19.7	25.3 (19.8)	88.2
Carbohydrate (g)	NA	NA	NA	349	379 (163)	0.8
**Micronutrients**						
Calcium (g)	334	418 (321)	76.1	227	335.5 (296.4)	89.6
Iron (mg)	11.3	13.7 (9.3)	78.0	5.2	6.8 (5.5)	94.6
Zinc (mg)	NA	NA	NA	4.2	5.1 (3.5)	87.8
Vitamin C (mg)	28.0	43.0 (48.0)	50.6	35.1	52.7 (71.6)	82.0
Vitamin B1 (mg)	1.1	1.3 (0.7)	16.1	1.2	1.3 (0.6)	45.4
Vitamin B2 (mg)	0.7	0.8 (0.3)	52.5	0.74	0.9 (0.7)	80.5
Niacin—Vitamin B3 (mg)	12.9	13.8 (6.3)	13.4	12.2	13.1 (6.1)	59.8
Vitamin B6 (mg)	NA	NA	NA	0.6	0.7 (0.4)	94.3
Folate total (µg)	109.0	129.0 (84.5)	72.0	104	128.8 (99.1)	96.9
Vitamin B12 (µg)	NA	NA	NA	0.0	0 (0.1)	98.2
Vitamin A (RAE) (µg)	124	291 (480)	83.2	9.0	27.6 (51.6)	98.2

^1^ Data are based on 24-h recalls of National Nutrition Monitoring Bureau in 2012 and do not include amounts consumed from supplements (such as calcium, iron, and folic acid). ^2^ Data are based on 24-h recalls from the baseline survey of the Alive & Thrive maternal nutrition study in 2016 in Unnao and Kanpur Dehat districts of Uttar Pradesh. These data do not include amounts consumed from supplements (such as calcium, iron, and folic acid). ^3^ Inadequate intakes were defined as <50% Recommended Dietary Allowances. ^4^ Inadequate intakes for macronutrients were defined based on the following Acceptable Macronutrient Distribution Ranges suggested by Institute of Medicine [19]: protein, 10–35%; fat, 20–35%; carbohydrate, 45–65% of total energy. Inadequate intakes for micronutrients were defined when mean intakes were below estimated average requirement [20].

**Table 2:** Factors associated with dietary intake among pregnant women, NFHS-4 data 2015–16.

	Pulses	Dairy	Meat/Fish/Eggs	Fruits	Dark Green Leafy Vegetables	Unhealthy Foods
**Household**	OR (95% CI)	OR (95% CI)	OR (95% CI)	OR (95% CI)	OR (95% CI)	OR (95% CI)
Socioeconomic status (ref: poorest)						
Poor	1.10 (1.00, 1.20)	1.34 *** (1.22, 1.48)	1.32 * (1.04, 1.68)	1.00 (0.92, 1.10)	1.65 *** (1.36, 2.00)	1.01 (0.90, 1.15)
Middle	1.07 (0.97, 1.18)	1.71 *** (1.54, 1.90)	1.91 *** (1.50, 2.43)	1.07 (0.98, 1.18)	2.12 *** (1.77, 2.55)	0.91 (0.80, 1.04)
Rich	1.08 (0.98, 1.20)	2.43 *** (2.19, 2.70)	1.72 *** (1.33, 2.22)	0.99 (0.89, 1.09)	2.84 *** (2.36, 3.42)	0.82 ** (0.72, 0.93)
Very rich	1.10 (0.99, 1.23)	3.71 *** (3.31, 4.15)	1.84 *** (1.43, 2.38)	1.05 (0.94, 1.17)	5.01 *** (4.17, 6.02)	0.73 *** (0.64, 0.84)
Household size (ref: ≤4)						
5–6 people	1.09 * (1.02, 1.18)	0.99 (0.92, 1.07)	0.73 *** (0.63, 0.85)	0.92 * (0.86, 0.99)	0.76 *** (0.69, 0.85)	1.05 (0.96, 1.15)
≥7 people	1.27 *** (1.18, 1.36)	0.97 (0.89, 1.04)	0.62 *** (0.53, 0.72)	1.01 (0.94, 1.08)	0.66 *** (0.59, 0.74)	1.10 * (1.01, 1.21)
Place of residence (ref: rural)	1.12 ** (1.03, 1.22)	1.66 *** (1.51, 1.82)	1.28 ** (1.09, 1.50)	1.11 * (1.02, 1.21)	2.74 *** (2.47, 3.04)	0.81 *** (0.73, 0.90)
Hindu religion (ref: other)	1.35 *** (1.24, 1.46)	1.49 *** (1.37, 1.62)	0.34 *** (0.30, 0.39)	1.02 (0.94, 1.11)	0.79 *** (0.71, 0.89)	1.14 ** (1.04, 1.26)
**Mother**						
Age, y	1.01 (1.00, 1.01)	0.99 * (0.98, 1.00)	0.99 (0.97, 1.00)	1.00 (0.99, 1.01)	0.99 * (0.98, 1.00)	1.00 (0.99, 1.01)
Early marriage	1.01 (0.95, 1.07)	0.85 *** (0.79, 0.92)	1.10 (0.95, 1.27)	1.05 (0.99, 1.12)	0.77 *** (0.69, 0.85)	1.03 (0.95, 1.12)
Education (ref: illiterate)						
Primary	1.03 (0.94, 1.14)	1.07 (0.96, 1.18)	1.96 *** (1.51, 2.53)	1.07 (0.98, 1.18)	1.57 *** (1.29, 1.90)	0.98 (0.87, 1.12)
Secondary	1.24 *** (1.14, 1.35)	1.61 *** (1.48, 1.75)	2.53 *** (2.05, 3.11)	1.28 *** (1.18, 1.38)	2.80 *** (2.41, 3.26)	0.97 (0.87, 1.07)
High school or higher	1.68 *** (1.48, 1.90)	2.98 *** (2.59, 3.42)	3.63 *** (2.81, 4.69)	1.30 *** (1.15, 1.47)	5.66 *** (4.72, 6.79)	0.91 (0.79, 1.06)
Scheduled caste/tribe/OBC (ref: general)	0.85 *** (0.79, 0.92)	0.79 *** (0.72, 0.86)	1.18 (1.00, 1.39)	0.95 (0.88, 1.02)	0.92 (0.81, 1.04)	0.98 (0.88, 1.08)

OR: odds ratio; OBC: other backward classes. * *p* < 0.5, ** *p* < 0.01, *** *p* < 0.001.

**Table 3:** Summary of enablers of and barriers to optimal diets from subnational data.

Author, Year	State	Study Objective	Design/Sample Size	Enablers	Barriers
Alive & Thrive (2016) [23]	Uttar Pradesh	To understand the factors that influence a mother’s diet and the feeding practices of her children.	In-depth interviews and observations (*N* = 360 PW and mothers), small group discussions (*N* = 218 husbands and mothers-in-law).	Most women were confident that they could incorporate green leafy vegetables, followed by lentils, milk, and milk products; over 40% were confident that they would be able to consume animal foods daily. The primary factors that would facilitate uptake of the recommended foods are cheaper price and support from family members.	The primary barriers preventing daily consumption of food from all food groups include affordability, availability, and food habits.
Andersen et al. (2003) [24]	Tamil Nadu	To describe how factors such as education level, economy, and folk dietetics influence PW’s food choice.	24-h dietary recall with weighing of foods and recipes of dishes (*N* = 30 PW).	Factors such as education level, family type, pregnancy number, and folk dietetics did not have a negative effect on food choices.	Eating customs and economic factors influenced women’s food choice negatively in relation to recommendations.
Catherin et al. (2015) [25]	Karnataka	To assess beliefs and practices regarding nutrition during pregnancy and lactation.	4 FGDs and 12 in-depth interviews	N/A	Avoidance of food items like ragi, papaya, mango and guava during pregnancy and reduced water consumption during the postnatal period. Beliefs like “casting an evil eye” or “color of the baby” had an influence on the food given to antenatal mother.
Chakrabarti et al. (2019) [26]	West Bengal	To examine food taboos in pregnancy and early lactation.	Qualitative with 4 FGDs Cross-sectional interview (*N* = 44 pregnant and lactating women)	N/A	Taboos on consumption of fruits (banana, papaya, jackfruit, coconut), vegetables (brinjal, leafy vegetables), meat, fish, and eggs during pregnancy to prevent miscarriage and fetal malformations and promote easy delivery. Taboos in the lactation period included avoidance of small fish, foods with multiple seeds, other "cold" foods, and fluid restriction in some areas.
Chakrabarti et al. (2019) [27]	India	To investigate coverage and equity of food supplementation for pregnant women.	*N* = 36,850 mother–child pairs in 2006 and 190,804 in 2016.	N/A	Supplementary food for PW increased between 2006 and 2016, from 19% to 53%. Key barriers to access food supplementation were being in the poorest quintiles, low schooling levels, and belonging to disadvantaged castes and tribes.
Craig et al. (2018) [28]	Tamil Nadu	To understand maternal dietary beliefs and practices.	Ethnographic study (*N* = 33).	Some women reported certain foods were beneficial during pregnancy, including apples, forest greens, egg, and various meats (chicken, mutton, rabbit, and deer).	Women avoided foods including fruit, animal products, tubers and root vegetables, legumes/pulses, and seeds during pregnancy. Most food avoidance was based on advice from elders and family members. Women reduced consumption of millets and switched to rice due to lack of availability.
Ganpule-Rao et al. (2020) [29]	Maharashtra	To assess food access and nutritional status among adolescents.	Cross-sectional, (*N* = 418).	With easier access to food, consumption of staple foods decreased and outside food increased.	N/A
Jhaveri et al. (2021) [30]	Uttar Pradesh	To identify key facilitators and barriers that affect behavioral adoption for diet diversity.	Qualitative in-depth interviews with 24 PW, 13 husbands, and 15 mothers-in-law.	High overall awareness and knowledge of dietary diversity recommendations among PW and family members. Family support to procure nutritious foods.	Structural opportunity barriers (financial strain, lack of food availability and accessibility) and individual barriers such as food preferences (likes and dislikes) and nausea prevented consistent behavioral change.
Kachwaha et al. (2020) [31]	Uttar Pradesh	To examine the cost and affordability of nutritious diets for households with PW.	24 market and 125 household surveys.	Home production had potential to reduce the cost of nutritious diets by 35%, subsidized grains by 19%, and supplementary food by 10%.	The nutritious diet was unaffordable for 75% of households given current income levels, consumption patterns, and food prices. Household income and dietary preferences, rather than food availability, were the key barriers to obtain nutritious diets.
Kehoe, S. H., et al. (2019) [32]	Maharashtra	To identify barriers and facilitators to fruit and vegetable consumption among WRA.	9 FGDs and 12 in-depth interviews	Women knew that fruit and vegetables were beneficial to health and wanted to increase the intake of these foods for themselves.	Potential barriers to fruit and vegetable consumption: (1) personal factors, (2) household dynamics, (3) social and cultural norms, (4) workload, (5) time pressures, (6) environmental factors, and (7) cost.
Lakshmi (2013) [33]	Andhra Pradesh	To examine the food preferences and restrictions practices by tribal PW women	In-depth interviews and direct observation. *N* = 600 PW aged 15–45.	Tribal women prefer iron-rich food during the antenatal period, believe it is food for growth and development of the fetus. Of the women, 60% believed nutrient-rich foods should be consumed during pregnancy.	Restrictions to consume raw papaya, sesame, coconut water, and fermented rice because they fear it may induce abortions. Of the women, 82% believed that some food items should be restricted for PW.
Mukhopadhyay and Sarkar (2009) [34]	Sikkim	To document pregnancy-related food practices and the social–cultural factors linked with them.	Cross-sectional study with mothers with child <1 year (*N* = 199).	Higher literacy and lower parity were associated with consuming special foods (milk, animal protein, pulses, green vegetables, and fruits) during pregnancy.	Taboos on different food categories, including milk, eggs, fish, meat, pulses, green vegetables, and fruits (perceived as hot and sour foods) during the postpartum were reported by 65% of mothers.
Nguyen et al. (2019) [21]	Uttar Pradesh	To understand the factors associated with consumption of diverse diets.	Cluster-randomized control trial with repeated cross-sectional surveys (*N* = 667 PW)	Higher dietary diversity and greater number of food groups consumed was associated with higher nutrition knowledge (OR = 1.2), receiving counseling (OR = 1.9), higher maternal education (OR = 1.1), and higher economic status (OR = 1.1).	Lower diet diversity and consumption of fewer number of food groups were associated with low caste status and higher parity (OR = 0.9).
Raghunathan et al. (2021) [35]	India	To estimate the cost of satisfying India’s national dietary guidelines and assess this diet’s affordability.	Nationally representative rural price and wage data	Nutritious diets became substantially more affordable over time.	Nutritious diets in 2011 were expensive relative to unskilled wages, constituting approximately 80–90% of female and 50–60% of male daily wages. Overall, estimate that 63–76% of the rural poor could not afford a recommended diet in 2011.
Rammohan et al. (2018) [36]	Uttar Pradesh	To assess the socioeconomic factors associated with dietary diversity among PW.	Cross-sectional study (*N* = 230)	Women with higher education and economic status were less likely to have low dietary diversity (OR = 0.4).	Dietary diversity was low among women who had no prior contact with a health professional or doctor (OR = 0.6), and those with parity of two or more (OR = 1.2).
Roshni (2019) [37]	Chhattisgarh	To assess feasibility to work with men and boys to improve the nutritional status of adolescent girls, pregnant women, and mothers of children <2 years.	FGDs with women, men, and adolescent boys and girls. In-depth interviews with girls and mothers. Key informant interviews with key stakeholders	N/A	Strong gendered roles inside and outside the household, social norms, reproductive health and nutrition behaviour and practice. Women had limited mobility, lower decision-making power. Women tend to eat the least amount of food and eat last in their family.
Sadhu et al. (2017) [38]	Rajasthan	To identify realistic and specific dietary advice/recommendations to address nutrient gaps for PW and lactating women.	Cross-sectional study (*N* = 2160)		Solid foods were specifically restricted during pregnancy. Maternal beliefs related to “hot” and “cold” foods relate to abortion and in causing difficulty in delivery of the baby. Women did not consume suggested foods due to lack of money and dislike of the prescribed food.
Sarkar, A., et al. (2020) [39]	Madhya Pradesh	To assess how underlying factors influence nutrition diversity and food security.	Cross-sectional study	Access to food and nutrition services (OR = 1.5), exposure to nutrition counselling (OR = 1.3), and hygiene practices (OR = 1.8) were associated with higher dietary diversity and food security.	Caste status negatively influences dietary diversity, especially among women and those with household food insecurity.
Sharma et al. (2020) [40]	Delhi, Karnataka, Bihar, Rajasthan	To explore dietary patterns and their determinants among PW and lactating women.	Cross-sectional study. (*N* = 476 PW and 446 lactating women).	Women who received nutrition advice had 2–3 times higher odds of consuming a nutritious “high-mixed vegetarian diet”.	Hindus and women who lived in rural areas had higher odds of consuming a poor “low-mixed vegetarian diet” and lower odds of consuming a nutritious “high-mixed vegetarian diet” (OR = 6.9).
Unisa et al. (2020) [22]	Bihar, Chhattisgarh, Odisha	To measure the dietary diversity of PW.	Cross-sectional study (*N* = 17,680 PW)	Having at least 6 years of education, belonging to a relatively rich household, and possessing a ration card increased mean dietary diversity.	N/A

Note: FGDs: focused group discussion; BMI: body mass index; N/A: not applicable; OR: odds ratio; PW: pregnant women; RDW: recently delivered women; WRA: women of reproductive age.

**Table 4:** Studies on program strategies in India to improve maternal diets.

Author, Year	State	Study Objectives	Intervention Strategies	Design/Sample Size	Key Findings
Sethi, V. et al. (2019) [45]	Andhra Pradesh, Telangana	To evaluate the maternal spot feeding programs in 2 Southern Indian states	Hot cooked meal	Cross-sectional surveys with PW and lactating women (*N* = 720); open-ended interviews (*N* = 252)	Average days of consumption of hot cooked meal in a month ranged from 17 to 22 days, against the targeted 25 days. Hot cooked meal enhanced high dietary diversity (≥6 food groups; 57–59%) and consumption of eggs and milk (74–96%) among pregnant and lactating women. Computed dietary energy and protein intake was higher on days when hot cooked meal was consumed compared with on days it was not consumed.
National Institute of Nutrition (2016) [46]	Andhra Pradesh	To assess the effect of hot cooked meal on nutritional status of pregnant and lactating women	Hot cooked meal	Ex-post quasi-experimental design; 24-h dietary recall (*N* = 516)	The per capita distribution of different foods and nutrients under the HCM program was lower than program norms with 102–130 feeding days for pregnant and lactating women against the target of 150 days. About 80% of women consumed meals and felt that quality of food was moderate to good. Consumption of all foods except cereals and nutrient intakes were lower than recommended levels.
Babu, G.R, et al. (2020) [47]	Karnataka	To estimate the impact of hot cooked meal on gestational weight gain and hemoglobin	Hot cooked meal	Mixed methods: quantitative surveys and qualitative in-depth interviews *N* = 1257 PW	Mothers who consumed HCM for >75 days had improved weight gain (average 10.27 kgs from the 1st to 3rd trimester) and an increase in hemoglobin (average 0.52% from the 1st to 3rd trimester).
Gupta et al. (2014) [48]	India	To assess potential of fermented foods in THR	THR	Lab-based methods	A Lactobacillus plantarum strain isolated from the dosa has been found to inhibit the growth of a range of food-borne pathogens. Thus, fermented foods have potential roles on gut health and nutrition status.
Raghunathan et al. (2017) [49]	Odisha	To study the effects of the Mamata conditional cash transfer scheme on use of maternal nutrition services and household food security	Cash transfer	Cross-section. household survey (*n* = 1161 mothers with children <2 years)	Receipt of payments from the Mamata scheme is associated with a decline of 0.84 on the Household Food Insecurity Access Scale. Nearly 60% of mothers enrolled in the scheme, and over 90% of those enrolled reported receiving money from the Mamata scheme. The key bottlenecks were transfers being smaller than expected and delays in payments to beneficiaries.
Aaron et al. (2016) [50]	Rajasthan	To assess household coverage of atta wheat flour, edible oil, and salt.	Food fortification	Cross-section. *N* = 4627 households (including caregiver and child <2 years)	Only 7% of the atta wheat flour was classified as fortifiable, and only 6% was actually fortified (mostly inadequately). For oil, almost 90% of edible oil consumed by households was classified as fortifiable, but only 24% was fortified. 66% of households used adequately fortified salt. The key bottleneck was most households (82%) reported purchasing whole grain and milling at home/local mills. A second major bottleneck was lack of monitoring and evaluation of compliance to fortification protocols.
Chakrabarti et al. (2019) [51]	Punjab, Tamil Nadu vs. Haryana, Andhra Pradesh, Karnataka	To assess impact of subsidized fortified wheat on anemia among PW	Food fortification	District panel. *N* = 10,186 Difference-in-difference analysis	In northern India, no impact on Hb or anemia reduction was found, as expected, given that the intervention targeted only non-poor households and demand for fortified wheat was low. In southern India, where intervention coverage was high, there was no impact on Hb but an impact on anemia reduction (8%), which was unexpected given low consumption of wheat in this predominantly rice-eating region.
Collison et al. (2015) [52]	Bihar	To explore the acceptability and utility of a low-cost and simple-to-use feeding toolkit of optimal dietary practices	BCC	16 FGDs and 8 key informant interviews; 14 days of user testing with 60 PW, lactating women, and RDW	After using the toolkit, the proportion of pregnant and breastfeeding women consuming an extra portion of food per day increased from 0% to 100%, and the number of meals taken per day increased from 2–3 to 3–4 meals. The toolkit, which is made of plastic, was well accepted by the community, although the communities recommended manufacturing the bowl and spoon in steel. Some mothers-in-law were not present during initial counseling, thus worried of the toolkit benefits and did not allow PW to use them. Some PW were hesitant to use the tools for fear that their morning sickness would intensify.
Garg and Kashyap (2006) [53]	Uttar Pradesh	To assess the effect of counseling on dietary intakes during pregnancy	BCC	Baseline and endline survey (*N* = 100 PW)	There was a significant increase in the amount of all food groups and nutrients consumed among those who received nutrition education post-intervention. However, the improvements still did not meet adequate intakes, except for vitamin A and C. Husbands and mothers-in-law played an important role in motivating women to adopt recommended behaviors.
Nguyen et al. (2021) [54]	Uttar Pradesh	To assess the impact of Alive & Thrive nutrition counseling interventions on maternal dietary diversity	BCC	Cluster-randomized control trial with repeated cross-sectional surveys (*N* = 660 PW)	Women in the intervention arm received more counseling on core nutrition messages (DID: 10–23 pp). Maternal food group consumption (∼4 food groups) and probability of adequacy of micronutrients (∼20%) remained low in both arms. Interventions showed modest impact on consumption of vitamin A-rich foods (10 pp, 11 g/d) and other vegetables and fruits (22–29 g/d). Factors explaining modest impacts on dietary diversity included limited resources and food preferences.
Shivalli et al. (2015) [55]	Uttar Pradesh	To examine the effectiveness of a BCC intervention on dietary intake during pregnancy.	BCC	Community-based quasi-experimental study with a control group (*N* = 86 PW)	The mean intake of protein increased by 1.8 grams in the intervention group and decreased by 1.8 grams in the control group. More than two-thirds of PW in the intervention group were taking one extra meal compared to only one-third in the control group. More than half of PW in both study groups decreased their dietary intake since conception.
Daivadanam et al. (2018) [56]	Kerala	To assess the effectiveness of a BCC intervention on dietary intake at the individual and household level	BCC	Community-based cluster-randomized controlled trial (*N* = 471)	There was significant, modest increase in fruit intake from baseline in the intervention arm (12.5%), but no significant impact of the intervention on vegetable intake over the control arm. Monthly household consumption of salt, sugar, and oil was greatly reduced in the intervention arm compared to the control arm, with the actual effect sizes showing an overall reduction by 45%, 40%, and 48%, respectively.
Vani Sethi et al. (2019) [57]	Delhi and Uttar Pradesh	To develop a maternal service package of interventions for mothers-to-be integrated in routine services at nutrition rehabilitation centers	BCC	427 mothers of inpatient children with severe acute malnutrition (SAM)	Universal interventions for all mothers not at nutritional risk include hospital diet, micronutrient supplementation and deworming, group-based nutrition education and counselling. Additional interventions for mothers at some nutritional risk include 15 minutes of individual, mother-focused tailoring to the mother’s specific nutritional risk. Mothers at severe nutritional risk got therapeutic foods (F-100). Evaluation was not done.
Beesabathuni et al. (2021) [58]	Madhya Pradesh	To test a poultry cooperative model to improve women empowerment, nutrition conditions, and SES	Nutrition-sensitive agriculture	Quasi-experimental study	Income increased by eight times per household, and more eggs became available in the community. Women and children now have access to an egg every other day
Kadiyala et. al (2021) [59]	Odisha	To test the effects of three nutrition-sensitive agriculture interventions on maternal diet diversity	Nutrition-sensitive agriculture	Four-arm, observer-blind, cluster-randomized trial (*N* = ~4736)	An increase in the proportion of mothers consuming at least five of ten food groups was seen in the nutrition-sensitive agriculture videos (adjusted RR 1.2) and nutrition-sensitive agriculture videos + participatory learning and action (RR: 1.30) groups compared with the control group, but not in the group that received only nutrition-specific videos (RR: 1.16).

Note: HCM: hot cooked meal; Hb: hemoglobin; BCC: behavior change communication; FDGs: focused group discussion; PW: pregnant women; RDW: recently delivered women; RR: risk ratio.

## Supplementary Materials

The following are available online at www.mdpi.com/article/10.3390/nu13103534/s1, Figure S1: Dietary intake among pregnant women in selected states.
