# Supplementary material for: Maternal Diets in India: Gaps, Barriers, and Opportunities
Source: Nutrients. 2021 Oct 9;13(10):3534. doi: 10.3390/nu13103534 (PMC8540854; doi:10.3390/nu13103534)
Supplement: Supplementary file 1 [file nutrients-13-03534-s001.zip › nutrients-1397891-supplementary.pdf]

Figure S1: Dietary intake among pregnant women in selected states[1]

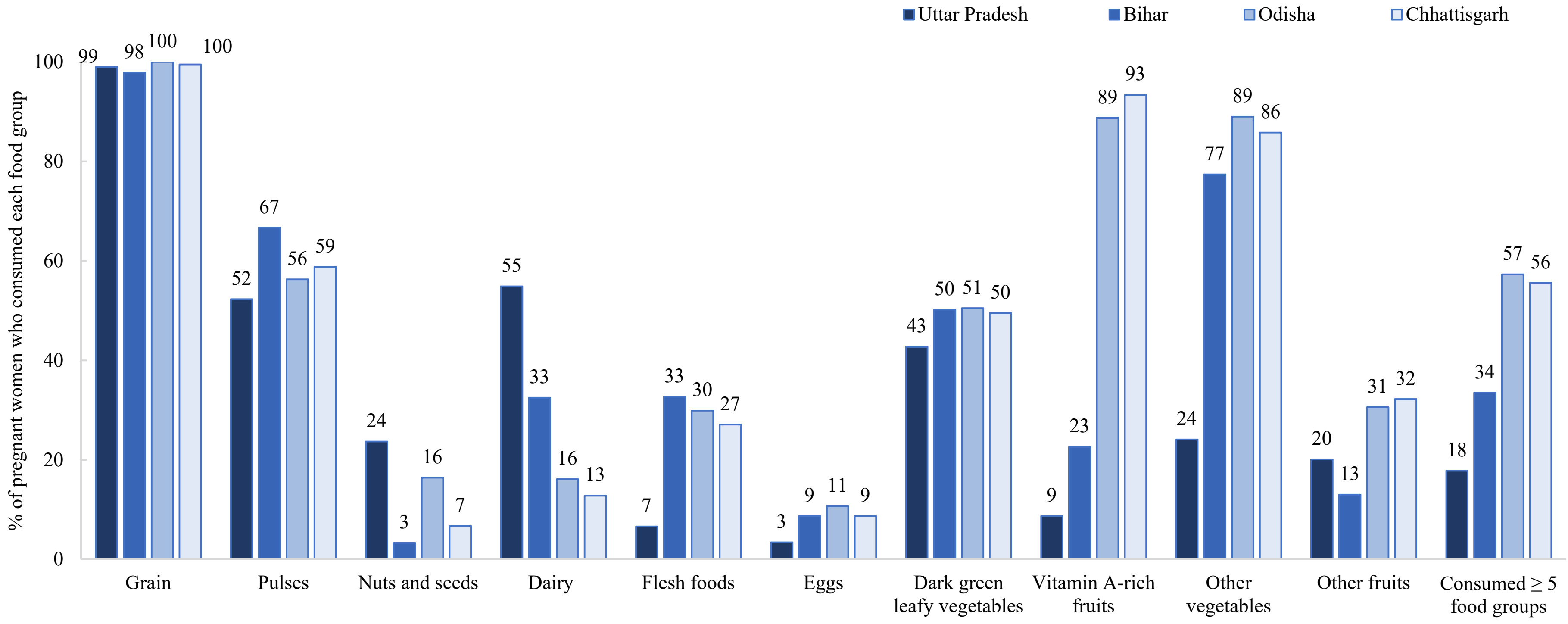

[1]Subnational data extracted from baseline survey of the Alive &Thrive maternal nutrition study in 2016 in Uttar Pradesh and baseline survey of the Swabhimaan programme in 2016 Bihar, Chhattisgarh and Odisha.
